# Therapeutic Effect of Chenodeoxycholic Acid in an Experimental Rabbit Model of Osteoarthritis

**DOI:** 10.1155/2015/780149

**Published:** 2015-10-11

**Authors:** Zhao-wei Yan, Ji Dong, Chen-hao Qin, Chun-yang Zhao, Li-yan Miao, Chun-yan He

**Affiliations:** ^1^Department of Clinical Pharmacology, The First Affiliated Hospital of Soochow University, Suzhou, Jiangsu 215006, China; ^2^Department of Clinical Laboratory, The Second Affiliated Hospital of Soochow University, Suzhou, Jiangsu 215004, China; ^3^Department of Radiology, The Second Affiliated Hospital of Soochow University, Suzhou, Jiangsu 215004, China

## Abstract

Osteoarthritis (OA) is a slowly progressive joint disease typically seen in middle-age to elderly people. At present, there is no ideal agent to treat OA. Chenodeoxycholic acid (CDCA) was a principal active constituent from animal bile. However, the therapeutic effect of CDCA on OA severity was largely unknown. The purpose of this study was to evaluate the therapeutic effect of intra-articular injection of CDCA in a rabbit OA model. OA was induced in experimental rabbits by anterior cruciate ligament transection (ACLT) and then rabbits were intra-articularly injected with CDCA (10 mg/kg or 50 mg/kg) once per week for 5 weeks. The results showed that CDCA significantly decreased cartilage degradation on the surface of femoral condyles, reducing the pathological changes of articular cartilage and synovial membrane by macroscopic and histological analysis. CDCA also significantly decreased bone destruction and erosion of joint evaluated by micro-CT. Furthermore, CDCA could markedly reduce the release of matrix metalloproteinase-1 (MMP-1), matrix metalloproteinase-3 (MMP-3), interleukin-1*β* (IL-1*β*), and prostaglandin E_2_ (PGE_2_) in synovial fluid. These observations highlight CDCA might be a potential therapeutic agent for OA.

## 1. Introduction

Osteoarthritis (OA) is a prevalent form of arthritic disease and a leading cause of physical disability in adult population [1–3]. It was generally considered a whole joint disease characterized mainly by cartilage destruction, subchondral bone sclerosis, osteophyte formation, and joint synovitis [4, 5]. Though much research has been performed, the concrete causes of OA remain unclear.

At present, OA progression is considered to be regulated largely by an excess of matrix metalloproteinases (MMPs), which contribute to the degradation of the extracellular matrix. Among these enzymes, MMP-1 and MMP-3 play important roles in OA progression by degrading the extracellular matrix [6–8]. Moreover, inflammatory mediators such as IL-1*β* and PGE_2_, have been implicated in the synovial inflammation and cartilage degradation in OA [9, 10]. Levels of IL-1*β* and PGE_2_ are increased in the synovial fluid and cartilage of OA patients, implying a role for IL-1*β* and PGE_2_ in the pathogenesis of OA [11].

The currently available pharmacological treatments for OA are effective only temporarily and might result in undesirable gastrointestinal, renal, and cardiovascular side effects [12–14]. There is an increasing interest in the use of natural compounds extracted from Traditional Chinese Medicine (TCM) for the treatment of OA because they are reported to demonstrate satisfactory clinical efficacy with minimal side effects, compared to routine pharmacological strategies [15]. Our group has previously reported that chenodeoxycholic acid (CDCA) was an important active constituent from animal bile and exhibited obviously inhibitory effect on MMPs in vitro [16, 17]. However, the effect of CDCA on OA severity in vivo has not yet been studied at present. In continuation of our previous study and development of a novel small molecule drug to treat OA, we elucidated the therapeutic effect of CDCA in an experimental rabbit model of OA for the first time in this paper.

## 2. Materials and Methods

### 2.1. Reagents

Chenodeoxycholic acid (CDCA, with its purity above 98%) and celecoxib (with its purity above 98%) were purchased from Sigma-Aldrich (St. Louis, MO, USA).

### 2.2. Experimental Animal Model and Drug Treatment

Forty SPF adult male white New Zealand rabbits weighing 3.0–3.5 kg were purchased from Shanghai SLAC Laboratory Animal Co. Ltd. (Shanghai, China). This experiment was carried out in accordance with the Chinese Guidelines for Animal Welfare and Experimental Protocol and approved by the Animal Care and Use Committee of Soochow University. The rabbits were placed in a left lateral position and the right operated legs were shaved and disinfected with betadine solution. Thirty-two of the 40 rabbits underwent anterior cruciate ligament transection (ACLT) on the right knee joints to induce OA [18] and were randomly divided into 4 groups (*n* = 8 per group). The other 8 rabbits (sham-operation group) received sham operations on the right knee joints, which involved opening the articular cavity and resuturing it without cutting the short anterior cruciate ligament. Postoperatively, the animals were permitted cage activity without immobilization. The rabbits were closely monitored for infections and other complications. Treatments began 4 weeks after surgery. Drugs were given once per week for 5 weeks. The rabbits in CDCA-treated groups were given intra-articular injections of CDCA (10 mg/kg or 50 mg/kg) in the knees operated on. The rabbits in celecoxib-treated group were intra-articularly injected with celecoxib (15 mg/kg) in the knees operated on. The rabbits in model group were intra-articularly injected with 0.5 mL vehicle alone in the knees operated on. In sham-operation group, no other procedures were conducted. All rabbits were sacrificed 7 days after the last injection.

### 2.3. Macroscopic Observations

After the treatment, the rabbits were sacrificed and femoral condyles were collected for macroscopic observation. The cartilage degradation on the surface of femoral condyles was observed under dissecting microscope and the degree of degradation was graded on a scale of 0–4 as follows: 0 = surface smooth with normal color; 1 = surface rough with minimal fibrillation or a slight yellowish discoloration; 2 = cartilage erosion extending into superficial or middle layers; 3 = cartilage ulceration extending into deep layers; 4 = cartilage depletion with subchondral bone exposed [18, 19]. The examination was performed by two independent observers who were kept unaware of the treatment groups.

### 2.4. Histological Examination

After macroscopic observations, isolated specimens were prepared for further histological analysis. The specimens were decalcified (10% EDTA), embedded in paraffin, cut into 5 *μ*m sections, and stained with hematoxylin and eosin (H&E). The histological evidence of articular cartilage was assessed according to the scoring system by Kikuchi et al. and eight parameters [20], namely, loss of superficial layer, erosion of cartilage, fibrillation and/or fissures, loss of proteoglycan, disorganization of chondrocytes, loss of chondrocytes, exposure of subchondral bone, and cluster formation. In addition, the histological evidence of synovial membrane was also assessed based on the four parameters [21, 22], namely, intimal hyperplasia, lymphocytic/plasmocytic infiltration, subintimal fibrosis, and vascularity. All sections were graded by two independent observers that were kept unaware of the treatment groups.

### 2.5. Micro-Computed Tomography (Micro-CT) Scanning

At the end of study, the right knee joints of experimental rabbits were subject to analysis using a SkyScan micro-CT apparatus, operating at a resolution of 35 *μ*m voxel size. Three-dimensional reconstructions were performed using NRECON software, and the images were further processed with CT-analyzer software. The projection images were reconstructed into three-dimensional images using NRECON software and CT-analyzer (both from SkyScan).

### 2.6. ELISA for Measurement of MMP-1, MMP-3, IL-1*β*, and PGE_2_ in Synovial Fluid

Synovial fluids were obtained from anesthetized animals before sacrifice. The samples were stored at −70°C until assayed. The levels of MMP-1, MMP-3, and IL-1*β* in synovial fluid were measured by ELISA kits (TSZ Scientific LLC, Framingham, MA, USA). The levels of PGE_2_ in synovial fluid were measured by ELISA kits (R&D Systems, Minneapolis, MN, USA). All the assays were performed in accordance with the manufacturer's instructions. All samples from animals in each experimental group were assayed in duplicate.

### 2.7. Statistics

Data were presented as the mean ± SEM. Statistical analyses were performed using SPSS 16.0. Statistical comparisons were performed using the Mann-Whitney *U* test. *P* < 0.05 was considered significant.

## 3. Results

### 3.1. Macroscopic Observations

**Figure 1:**
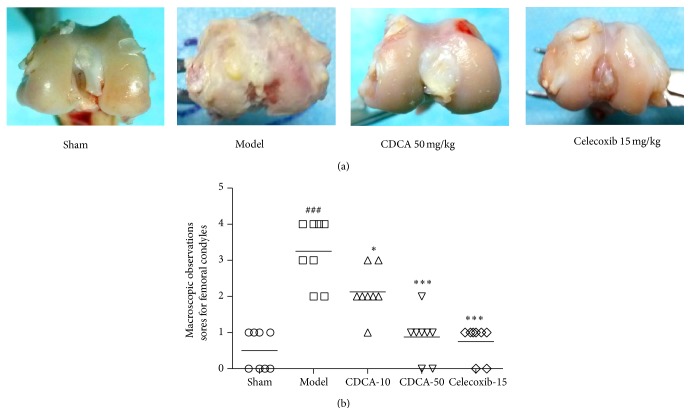
(a) Representative macroscopic observations images of femoral condyles are shown. (b) Macroscopic observations sores for femoral condyles in the five groups of experimental rabbits. Results are presented as individual data points and the median for each group is indicated by a horizontal bar. ^###^ *P* < 0.001, compared with the sham-operation group; ^∗^ *P* < 0.05, ^∗∗∗^ *P* < 0.001, compared with the model group. Similar results were observed in two separated experiments.

As shown in Figure 1(a), the cartilage on the femoral condyles in the sham-operation group was macroscopically normal, with a smooth, glistening surface, and no cartilage defect or osteophyte was observed. In the model group, general characteristics of OA, including erosion and osteophyte formation, were seen on the side of the femoral condyles after surgery. In the treatment groups, less bone wear was observed after CDCA or celecoxib treatment, compared with the model group. Accordingly, as scored by macroscopic observations, CDCA or celecoxib treatment could significantly decrease the degree of cartilage degradation (Figure 1(b)).

### 3.2. Histological Findings

**Figure 2:**
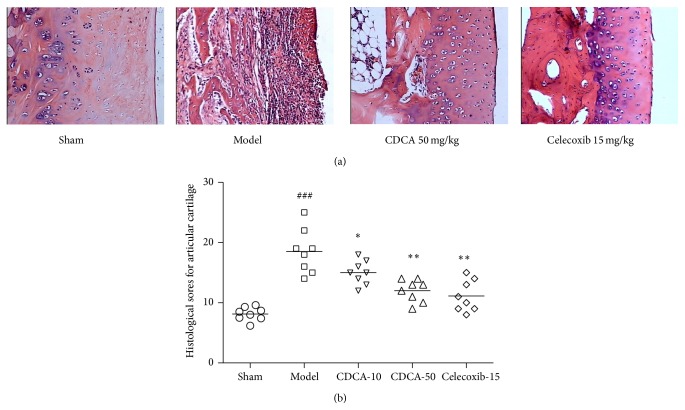
(a) Representative hematoxylin-eosin (HE) stained sections of articular cartilage are shown. Magnification 100x. (b) Histological sores for articular cartilage in the five groups of experimental rabbits. Results are presented as individual data points and the median for each group is indicated by a horizontal bar. ^###^ *P* < 0.001, compared with the sham-operation group; ^∗^ *P* < 0.05, ^∗∗^ *P* < 0.01, compared with the model group. Similar results were observed in two separated experiments.

As shown in Figure 2(a), the sham operation of rabbits revealed no significant histological changes in the articular cartilages, whereas the model group and other treated groups developed different degrees of OA-like degenerative changes. A significant decrease in the severity of cartilage degradation was observed in CDCA-treated groups, particularly in the 50 mg/kg group. Histological sores in the CDCA-treated and celecoxib-treated group were significantly lower than in the model group (Figure 2(b)).

**Figure 3:**
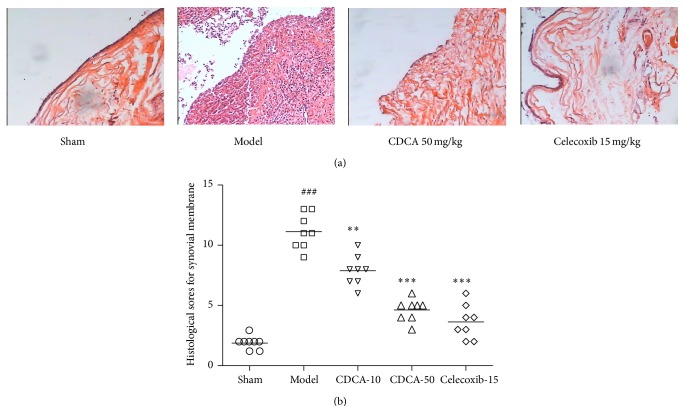
(a) Representative hematoxylin-eosin (HE) stained sections of synovial membrane are shown. Magnification 100x. (b) Histological sores for synovial membrane in the five groups of experimental rabbits. Results are presented as individual data points and the median for each group is indicated by a horizontal bar. ^###^ *P* < 0.001, compared with the sham-operation group; ^∗∗^ *P* < 0.01, ^∗∗∗^ *P* < 0.001, compared with the model group. Similar results were observed in two separated experiments.

In synovial membrane tissues, the model group showed a similar degree of thickened synovium associated with hyperplasia, hypotrophy, inflammatory cell infiltration, and vascularization (Figure 3(a)). The synovium changes in the CDCA-treated groups showed significant alleviation compared to model groups (Figure 3(b)).

### 3.3. Micro-CT Analysis

Micro-computed tomography (micro-CT) analysis is a powerful tool for the evaluation of bone tissue because it provides access to the 3D microarchitecture of the bone. It is a nondestructive imaging technique that can be widely used to track changes in subchondral bone structure in drug intervention studies designed to treat the progression of OA [23, 24].

**Figure 4:**
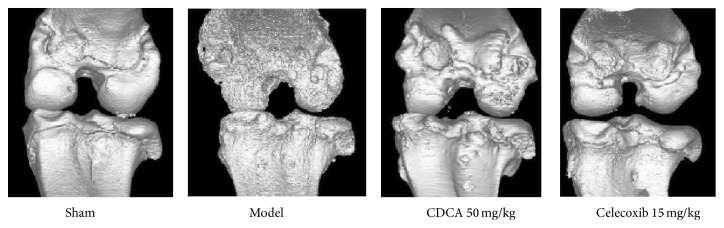
Representative micro-CT images of rabbit knee joints are shown.

In order to observe the bone changes of knee joints after CDCA treatment, micro-CT analysis was performed on OA rabbit treated with CDCA, celecoxib, and vehicle in this study. As shown in Figure 4, the bone contact was high and the surface of knee joints was smooth in the sham-operation group. In contrast, obvious bone destruction and erosion of joint was observed in the model group. The CDCA- (50 mg/kg) and celecoxib- (15 mg/kg) treated groups markedly prevented bone erosions at the knee joints; no obvious osteophyte formation was observed. The results suggested that CDCA treatment was an effective therapeutic approach to reduced joint destruction in OA rabbits.

### 3.4. Effects of CDCA on the Levels of MMP-1, MMP-3, IL-1*β*, and PGE_2_ in Synovial Fluid from Knee Joints Were Measured by ELISA

**Figure 5:**
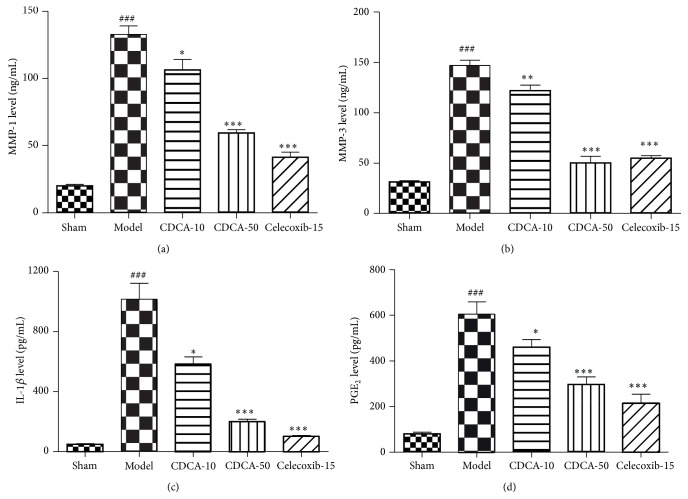
The levels of MMP-1 (a), MMP-3 (b), IL-1*β* (c), and PGE_2_ (d) in synovial fluid from knee joints were measured by ELISA. Each reported value is a mean ± SEM (*n* = 8); ^###^ *P* < 0.001, compared with the sham-operation group; ^∗^ *P* < 0.05, ^∗∗^ *P* < 0.01, and ^∗∗∗^ *P* < 0.001, compared with the model group. Similar results were observed in two separated experiments.

Compared to sham-operation group, model group showed higher level of MMP-1 and MMP-3 in synovial fluid (*P* < 0.001) (Figures 5(a) and 5(b)). The levels of MMP-1 and MMP-3 in synovial fluid were significantly decreased by CDCA treatment in a dose-dependent manner compared with those in model group. Moreover, the inflammatory mediators involved in cartilage destruction, such as IL-1*β* and PGE_2_, were also significantly inhibited by CDCA treatment (Figures 5(c) and 5(d)). The capability of CDCA to reduce the release of MMP-1, MMP-3, IL-1*β*, and PGE_2_ in synovial fluid was similar to the effect of celecoxib.

## 4. Discussion

Osteoarthritis (OA) is a prevalent form of arthritic disease and a leading cause of physical disability in adult population [1–3]. It was generally considered a whole joint disease characterized by cartilage destruction, subchondral bone sclerosis, osteophyte formation, and joint synovitis [4, 5]. At present, nonsteroidal anti-inflammatory drugs (NSAIDs), corticosteroids, and hyaluronan have been clinically used for the treatment of OA in the clinic. However, they fail to reverse cartilage damage, always resulting in undesirable cardiovascular, renal, and gastrointestinal side effects [14]. Thus, there is a continuing need for novel better agents with which to treat OA.

CDCA was an important active constituent from animal bile, which has been reported for the treatment of cardiovascular, cancer, respiratory, liver, and gallbladder diseases [25, 26]. However, little is known about its possible use in the treatment of OA. Our group has previously reported that CDCA exhibited obviously inhibitory effect on MMPs in vitro [16, 17]. In continuation of our previous study and search for a novel small molecule drug to treat OA, we elucidated the beneficial effect of CDCA on an OA model in rabbits for the first time in this paper.

In our study, we investigated the protective effect of CDCA against cartilage degradation in a rabbit OA model induced by ACLT, which is the most commonly used method of identifying OA disease-modifying therapies by intra-articular administration. The results showed that CDCA attenuated the severity of OA by reducing macroscopic observations sores for femoral condyles and histological sores for articular cartilage and synovial membrane (Figures 1–3). CDCA also significantly decreased bone destruction and erosion of joint evaluated by micro-CT (Figure 4). Notably, CDCA significantly reduced the degree of OA-like lesions at a dose of 50 mg/kg.

The matrix metalloproteinases (MMPs) have been considered the important enzymes responsible for degradation of aggrecan and collagens in cartilage [6, 7]. Many research groups have demonstrated that expression of several MMPs was elevated in synovial tissues and cartilage of OA patients [27]. MMP-1 belongs to collagenase subgroup in MMP family. It is able to cleave the triple helical chains of type II collagen in articular cartilage and play an important role in abnormal collagen turnover in OA [28–30]. MMP-3 also may have utility as a prognostic biomarker of OA progression. Patients with higher MMP-3 levels are more likely to suffer from progression of OA over a 30-month period than patients in the lower tertile [31]. Therefore, effects of CDCA on the levels of MMP-1 and MMP-3 in synovial fluid from rabbit knee joints were measured by ELISA in this paper. As shown in Figures 5(a) and 5(b), CDCA significantly decreased the levels of MMP-1 and MMP-3 in synovial fluid in a dose-dependent manner.

It is well established that many inflammatory mediators, such as IL-1*β* and PGE_2_, have been implicated in the synovial inflammation and cartilage degradation in OA [9, 10]. Levels of IL-1*β* and PGE_2_ are increased in the synovial fluid and cartilage of OA patients, implying a role for IL-1*β* and PGE_2_ in the pathogenesis of OA [11]. In this study, the levels of IL-1*β* and PGE_2_ in synovial fluid were also measured. Our results indicate that both IL-1*β* and PGE_2_ were significantly inhibited in the CDCA-treated group (Figures 5(c) and 5(d)). The capability of CDCA to reduce the release of MMP-1, MMP-3, IL-1*β*, and PGE_2_ in synovial fluid was similar to the effect of celecoxib.

Taken together, these results confirm the therapeutic potential of CDCA for treatment of OA. However, further studies are needed to clarify the exact mechanism of action of CDCA in the future.

## 5. Conclusions

In this study, we evaluated the therapeutic effect of intra-articular injection of CDCA in a rabbit OA model for the first time. The results showed that CDCA significantly attenuated the severity of OA as determined by macroscopic, histological, and micro-CT analysis. Furthermore, CDCA could markedly reduce the release of MMP-1, MMP-3, IL-1*β*, and PGE_2_ in synovial fluid. Therefore, highlighting CDCA might be a potential therapeutic agent for OA.
